# Supplementary material for: Clinical Interest of Serum Alpha-2 Macroglobulin, Apolipoprotein A1, and Haptoglobin in Patients with Non-Alcoholic Fatty Liver Disease, with and without Type 2 Diabetes, before or during COVID-19
Source: Biomedicines. 2022 Mar 17;10(3):699. doi: 10.3390/biomedicines10030699 (PMC8945355; doi:10.3390/biomedicines10030699)
Supplement: Supplementary file 1 [file biomedicines-10-00699-s001.zip › biomedicines-1619639-supplementary.pdf]

TABLE OF CONTENT

Supplementary File S1. Coauthors and Supplementary methods.

A. **FLIP consortium and FibroFrance group**
B. **Quid-Nash consortium**

Supplementary Figure S1: A2M in NAFLD-Biopsied patients with NASH

Supplementary Figure S2: ApoA1 NAFLD-Biopsied patients with significant NASH

Supplementary Figure S3: ApoA1 NAFLD-Biopsied patients with significant steatosis

Supplementary Figure S4: ApoA1 in NAFLD-Biopsied patients with obesity

Supplementary Figure S5: A2M in NAFLD-Biopsied patients without obesity

Supplementary Figure S6: A2M in NAFLD-Biopsied patients with obesity

Supplementary Figure S7: ApoA1 in NAFLD-Biopsied patients without obesity

Supplementary Figure S8: Hapto NAFLD-Biopsied patients without obesity

Supplementary Figure S9: Hapto in NAFLD-Biopsied patients with obesity

Supplementary Figure S10: Univariate correlations between three proteins and histological features in not-obese et obese male.

Supplementary Figure S11: Univariate correlations between three proteins and histological features in not-obese et obese female.

Supplementary Figure S12: Variation of the serum protein levels according to BMI for patients <50 years old

Supplementary Figure S13: Variation of the serum protein levels according to BMI for patients >=50 years old

Supplementary Table S1: T2D multivariate association with proteins and fibrosis.

Supplementary Table S2. T2D multivariate association with proteins and NASH.

Supplementary Table S3. T2D multivariate association with proteins and steatosis.

Supplementary File S1. Coauthors and Supplementary methods.

NAFLD-Biopsied-Population

A. FLIP consortium and FibroFrance group

The FLIP partners' consortium

Vlad Ratziu, Thierry Poynard: Assistance Publique Hopitaux de Paris, Groupe Hospitalier Pitie Salpetriere, Sorbonne University, Paris, France; Jean-Marie Castille, Yen Ngo: Biopredictive SAS, Paris, France; Tania Langon: Alma Consulting Group SAS,Paris, France; Chris Day, Dina Tiniakos: University of Newcastle, Newcastle, UK; Debbie Lawlor: University of Bristol, Bristol, UK; Giulio Marchesini: Universita di Bologna, Bologna, Italy; Fabio Marra: Università di Firenze, Firenze, Italy; Elisabetta Bugianesi: Università degli studi di Torino, Torino, Italy; Stefano Bellentani: Università di Modena e Reggio Emilia, Modena, Italy; Jean-Francois Dufour: Université de Berne, Berne, Switzerland; Manuel Romero Gomez: Servicio Andaluz de Salud, Spain; Thorkild Sørensen: Bispebjerg hospital, Region Hovedstaden, Bispebjerg, Dutch; Claudio Tribelli: Fondazione Italiana Fegato-Fonlus, Milano, Italy; Samuele De Minicis: Università di Ancona, Ancona, Italy; Michael Trauner: Medizinischen Universitaet, Wien, Austria; Claudia Oliveira: University of São Paulo School of Medicine, São Paulo, Brazil.

The FLIP pathology consortium

Pierre Bedossa: Assistance Publique-Hôpitaux de Paris, hôpital Beaujon, University Paris-Diderot, Paris, France; Alastair D. Burt: School of Medicine, University of Adelaide, Adelaide, Australia; Annette S.H. Gouw: Department of Pathology & Medical Biology, University Medical Center Groningen, Groningen, The Netherlands; Carolin Lackner: Institute of Pathology, Medical University of Graz, Graz, Austria; Peter Schirmacher: Institut Für Pathologie, Universitätsklinikum Heidelberg, Heidelberg, Germany; Luigi Terracciano: Institute of Pathology, University Hospital, Basel, Switzerland; Dina Tiniakos: Medical School, National and Kapodistrian University of Athens, Greece and Medical School, Newcastle University, Newcastle-upon-Tyne, UK; J. Brain: Medical School, Newcastle University, Newcastle-upon-Tyne, UK; Yvonne Bury: Royal Victoria Infirmary, Hospitals Foundation Trust, Newcastle upon Tyne, UK; Daniela Cabibi: University of Palermo, Palermo, Italy; Frederic Charlotte: Assistance Publique Hôpitaux de Paris, Groupe Hospitalier Pitié-Salpêtrière; Luisa Losi: Modena University Hospital, Modena, Italy; Matteo Montani: Institute of Pathology, University of Bern, Bern, Switzerland; María Jesus Pareja: Hospital Universitario de Valme, Sevilla, Spain; Dominique Wendum: Assistance Publique-Hôpitaux de Paris, Hopital St Antoine Sorbonne University; Fritz Wrba: Medical University of Vienna, Vienna, Austria; Marianne Ziol: Assistance Publique-Hôpitaux de Paris, Hôpital Jean Verdier, Universite Paris 13, Bobigny, France; Vlad Ratziu: Assistance Publique Hôpitaux de Paris, Hôpital Pitié Salpêtrière, Sorbonne University, Paris, France.

The FibroFrance group

Thierry Poynard, Vlad Ratziu, Dominique Thabut, Joseph Moussalli, Pascal Lebray, Marika Rudler, Francoise Imbert Bismuth, Frederic Charlotte, Olivier Rosmorduc, Yvon Calmus: Anti-Fibrosis Center, Assistance Publique Hôpitaux de Paris, Groupe Hospitalier Pitié-Salpêtrière, Sorbonne University, Paris, France; Agnes Hartemann, Sophie Jacqueminet: Diabetes Center, Assistance Publique Hôpitaux de Paris, Groupe Hospitalier Pitié-Salpêtrière, Sorbonne University, Paris, France; Eric Bruckert, Philippe Giral: Lipid Center, Assistance Publique Hôpitaux de Paris, Groupe Hospitalier Pitié-Salpêtrière, Sorbonne University, Paris, France;

Sylvie Naveau, Gabriel Perlemuter: Alcoholic liver Disease Center, Assistance Publique Hôpitaux de Paris, Antoine Beclere Hospital, Clamart, France; Brigitte Varsat: Prevention Centers of Caisse Primaire Assurance Maladie, Paris, France; Anne Mercadier: Transfusion Unit, Assistance Publique Hôpitaux de Paris, Groupe Hospitalier Pitié-Salpêtrière, Sorbonne University, Paris, France

Ethics.

For all clinical studies and the integrated present study, all authors had access to the study data and reviewed and approved the final manuscript and all patients provided written informed consent. All these studies were approved by the ethics committee at each participating institution and were performed according to good clinical practice and the Declaration of Helsinki.

Patients from the "Groupe Hospitalier Pitié-Salpêtrière cohort", Construction-1 (*Poynard T, Ratziu V, Naveau S, et al. The diagnostic value of biomarkers (SteatoTest) for the prediction of liver steatosis. Comp Hepatol 2005; 4:10.)*, Validation-1 (*Munteanu M, Tiniakos D, Anstee Q, et al. Diagnostic performance of FibroTest, SteatoTest, and ActiTest in patients with NAFLD using the SAF-score as histological reference. Aliment Pharmacol Ther 2016; 44:877–889.*), Validation-2 (*Poynard T, Lassailly G, Diaz E, et al. Performance of biomarkers FibroTest, ActiTest, SteatoTest, and NashTest in patients with severe obesity: meta-analysis of individual patient data. PLoS One. 2012;7:e30325.*), Target-1 and Target-3 (*Jacqueminet S, Lebray P, Morra R, et al. Screening for liver fibrosis by using a noninvasive biomarker in patients with diabetes. Clin Gastroenterol Hepatol. 2008;6:828-31.*) belong to FIBROFRANCE, a program organized in 1997 (Clinical registry number: NCT01927133; CPP-IDF-VI, 10-1996-DR-964, DR-2012-222). The protocol was approved by the institutional review board, regulatory agency and performed in accordance with principles of Good Clinical Practice. All patients provided written informed consent before entry. A subset of patients of Construction-1 with chronic hepatitis C, belong to a Schering Plough phase-3 randomized open-label trial performed at 62 centers in Europe, Canada, Argentina, and the USA. All patients provided written informed consent, and the protocol was approved by each center's institutional ethics committee. (*Manns MP, McHutchison JG, Gordon SC, et al. PEG-Interferon alfa-2b in combination with ribavirin compared to interferon alfa-2b plus ribavirin for initial treatment of chronic hepatitis C. Lancet 2001;358:958-965.*) A subset of patients of Validation-1 cohort not included in the FIBROFRANCE project, are part of the FLIP project for which a written patient consent for routine liver biopsy and data collection was obtained from each subject before inclusion. This epidemiological, non-interventional study was exempt from IRB review.

Target-2 subjects were consecutive subjects, forty years of age or older, who were seen for a free screening program in two French Social Security health examination centers. All procedures were performed in accordance with the current revised guidelines of the Declaration of Helsinki, approved by the ethical committee of Groupe Hospitalier Pitié Salpêtrière and all investigated participants gave informed signed consent.

Target-4 subjects were at risk of metabolic liver disease and required routine FibroTest without any interventional study, and not requiring IRB. Data were anonymized and analyzed for epidemiological purposes.

Histological references.

All biopsies were scored by experienced pathologists, blinded to historical biopsy reports, tests results, and other clinical data.

Construction-1, and Validation-1 (without hepatitis C).

The goal of the SAF scoring system, specific for NAFLD features, was to find a compromise between the development of a simple, easy to apply system to obtain a clear diagnosis in individual patients, even when performed by nonspecialists, and of a more reliable and discriminating system for therapeutic trials or to assess the diagnostic performance of biomarkers. A FLIP histopathology consortium of eight members developed the FLIP algorithm, a diagnostic tool for the diagnosis and staging of severe forms of NAFLD. According to the combination of each semi-quantification of the three elementary features of NAFLD using the SAF score for steatosis, inflammatory activity and fibrosis respectively. The steatosis score (S) assesses the quantities of large or medium-sized lipid droplets, with the exception of foamy microvesicles, and rates them from 0 to 3 (S0: <5%; S1: 5–33%, mild; S2: 34–66%, moderate; S3: >66%, marked). Activity grade (A, from 0 to 4) is the unweighted addition of hepatocyte ballooning (0–2) and lobular inflammation (0–2). Cases with A0 (A = 0) had no activity; A1(A = 1) had mild activity; A2 (A = 2) moderate activity; A3 (A = 3) severe activity and A4 (A = 4) had very severe activity. Fibrosis stage (F) was assessed using the score described by25 as follows: stage 0 (F0) = none; stage 1 (F1) = 1a or 1b perisinusoidal zone 3 or 1c portal fibrosis; stage 2 (F2) = perisinusoidal and periportal fibrosis without bridging; stage 3 (F3) = bridging fibrosis and stage 4 (F4) = cirrhosis (File S1). To reduce interobserver variability and homogenize the reading using the new SAF-FLIP histological classification, we used only reports reviewed by members of the FLIP Pathology Consortium (DT and PB for the FLIP subpopulation and FC for the FibroFrance subpopulation).

SAF, METAVIR scoring systems and pre-determined cutoffs

**SAF scoring system**		**METAVIR**
**Classes**	**Definition**	**Definition**
**Fibrosis**		
F0	None	None
F1	Perisinusoidal or portal	Portal fibrosis
F2	Perisinusoidal and portal without bridging	Few septa
F3	Bridging	Many septa
F4	Cirrhosis	Cirrhosis
**Activity**		
A0	Ballooning + lobular inflammation =0	No activity
A1	Ballooning + lobular inflammation =1	Minimal activity
A2	Ballooning + lobular inflammation=2	Moderate activity
A3	Ballooning + lobular inflammation =3	Severe activity
A4	Ballooning + lobular inflammation =4	No A4 class
**Steatosis**		
S0	<5%	<5%
S1	5%-33%	>5-33%
S2	>33-66%	>33%
S3	>66%	>66%

References:

SAF scoring system:

- *Kleiner DE, Brunt EM, Van Natta M, et al. Design and validation of a histological scoring system for nonalcoholic fatty liver disease. Hepatology 2005;41:1313-132120.*
- *Bedossa P, Poitou C, Veyrie N, et al. Histopathological algorithm and scoring system for evaluation of liver lesions in morbidly obese patients. Hepatology. 2012;56:1751-9.*

The histological check-list of SAF was from NASH-CRN (Kleiner et al) with a few additional comments. Among major features, ballooning was graded from 0 to 2 (0: normal hepatocytes with cuboidal shape and pink eosinophilic cytoplasm; 1: presence of clusters of hepatocytes with a rounded shape and pale cytoplasm usually reticulated. Although shape is different, size is quite similar to that of normal hepatocytes; 2: same as grade 1 with some enlarged hepatocytes, at least 2-fold that of normal cells). Lobular inflammation was defined as a focus of two or more inflammatory cells within the lobule. Foci were counted at 20x magnification (0: none; 1: ≤2 foci per 20x; 2: >2 foci per 20x). There was no change in the definition of hepatocellular ballooning proposed by the NASH-CRN, but reference was added to the size and shape of hepatocyte for clarity and the same number of scales (0-2) was used for both lesions.

METAVIR scoring system

- *Bedossa P, Poynard T. An algorithm for the grading of activity in chronic hepatitis C. The METAVIR Cooperative Study Group. Hepatology. 1996;24:289-93.*
- *Poynard T, Imbert-Bismut F, Munteanu M, et al. Overview of the diagnostic value of biochemical markers of liver fibrosis (FibroTest, HCV FibroSure) and necrosis (ActiTest) in patients with chronic hepatitis C. Comp Hepatol. 2004;23;3-8.*

B. NAFLD-Biopsied Quid-Nash

Project Members of the Quid-Nash Consortium involved in WP6:
Clinicians: Dominique Valla, Laurent Castera, Beaujon hospital, APHP, Paris, France; Thierry Poynard Groupe Hospitalier Pitié-Salpêtrière, APHP, Paris, France ; Stanislas Pol, Jean-Francois Gaultier, Lariboisière hospital, APHP ; Christian Boitard, Etienne Larger Cochin hospital, APHP, Paris, France.
Pathologists: Pierre Bedossa, Valérie Paradis, Beaujon hospital, APHP, Paris, France ; Benoit Terris Cochin hospital, APHP, Paris, France. **Radiologists:** Jean-Michel Correas, Necker hospital, APHP, Paris, France; Valérie Vilgrain, Beaujon hospital APHP, Paris, France. **Biochemists and biologists:** Valentina Peta, BioPredictive, Paris, France; Béatrice Parfait, Centre de Resources Biologiques Cochin hospital APHP, Paris, France. **Statisticians, Clinical Research, Data manager:** Cédric Laouénan, Jimmy Mullaert, Nathalie Gault, Estelle Marcault, Pauline Manchon, Nassima Si Mohammed, Bichat hospital APHP and Lariboisière Hospital APHP, Paris, France; Olivier Deckmyn, BioPredictive, Paris, France ; Mark Ibberson Swiss Institute BioInformatics SIB, Switzerland. **Administration:** Angélique Brzustowski Hopital Beaujon APHP, Paris, France ; Fabienne Drane, Jean Marie Castille Biopredictive, Paris, France.

Quid-Nash Project

Non-alcoholic steatohepatitis (NASH) is a chronic liver disease particularly frequent and severe in patients with type 2 diabetes (T2DM) in whom it cannot be recognized only by means of needle liver biopsy, a heavy and costly procedure poorly tolerated and accepted.
The main aim of the QUID-NASH program is to develop a virtual liver biopsy able to replace the current liver needle biopsy for the diagnosis and staging of NASH in patients with T2DM.

This first aim also includes a validation of diagnostic blood tests recently patented by BioPredictive. The QUID-NASH research program has been set up by a consortium combining teams from the following partners Inserm, Assistance Publique-Hôpitaux de Paris, Universities Paris-Descartes and Paris-Diderot, Commissariat à l'Energie Atomique (CEA), Laboratoires Servier and BioPredictive, Inserm being the leading partner.

The consortium is co funded by the partners (contributing up to 16 million euros) and, through the 3rd call "Recherche Hospitalo-Universitaire (RHU)", the French State (9 million euros). The main aim of the QUID-NASH program is to develop a virtual liver biopsy (non-invasive) for the diagnosis and staging of NASH (Non-Alcoholic SteatoHepatitis) in patients with type 2 diabetes (T2D). This first aim also includes a validation of diagnostic blood tests recently patented by BioPredictive. Second aims of the project consist in increasing our knowledge of the NASH pathology with the identification of new metabolic pathways, drug targets as well as predictive animal models (mouse and mini pig).

The clinical study involves 600 type 2 diabetic patients in whom a liver biopsy is needed as part of standard of care. Main implicated teams include diabetology in Lariboisière and Cochin hospitals, Hepatology in Beaujon and Cochin, Imaging in Necker Beaujon and Physics for Medicine Paris. The data collected (imaging, deep clinico-biological phenotyping, different atomic approaches, extracellular vesicles and immune blood cell profiling) will be exploited through comprehensive and deep bioinformatics and biostatistics involving the Swiss Institute of Bioinformatics (SIB) as well as Bichat biostatistical teams. Results are expected to be delivered in December 2022.

In a translational approach, parallel studies will be performed in animals with one aim being to speed up the development of active drugs for NASH. The implicated teams are part of Institut Cochin, CRI Paris Montmartre, Physics for Medicine Paris and Laboratoires Servier. The clinical and preclinical studies will allow collecting biological samples (blood, liver, urine, feces) as well as images that will be available (associated to phenotypic data) for additional, independent studies.

Supplementary Figure S1: A2M in NAFLD-Biopsied patients with NASH

Impact of T2D on A2M concentration in NAFD-Biopsied patients with NASH A2A3 (n=639). Normal serum means values by age and gender (dashed orange lines), with 95% confidence intervals (light gray ribbon). Three vertical dotted lines mark the 4 age groups (before 25, between 25 and 50, between 50 and 75 and above 75 years old). Each point is a protein patient value. The red curve is a Loess regression of the median of A2M values, along with its 95% confidence interval (darker gray). For each age group, the mean difference (%95CI) between patient protein value and the expected normal value (for its age and gender), with its significance p-value is displayed on top of figure. The significance between the non-T2D vs. T2D patients is displayed in red between the 2 panels.

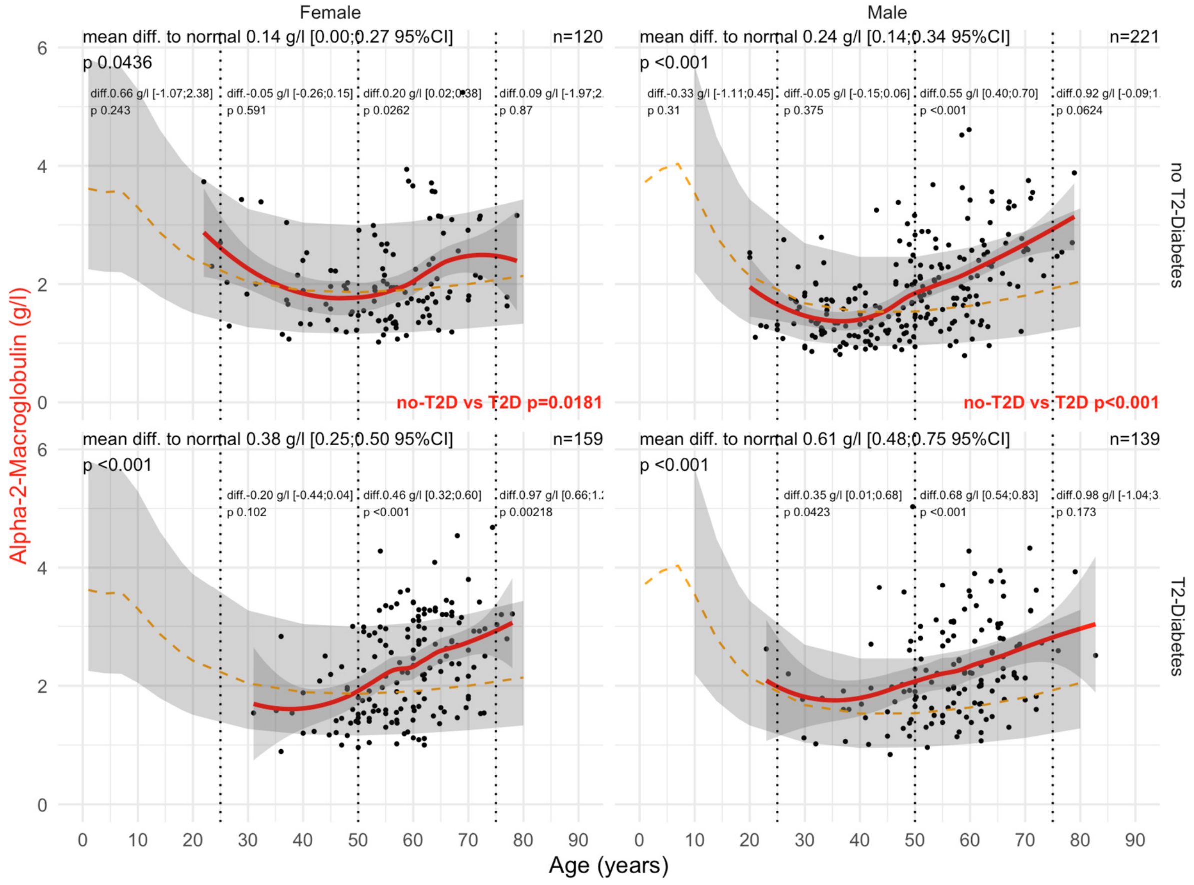

Supplementary Figure S2: ApoA1 NAFLD-Biopsied patients with significant NASH

Impact of T2D on ApoA1 concentration in NAFD-Biopsied patients with significant NASH A2A3 (n=639). Normal serum means values by age and gender (dashed orange lines), with 95% confidence intervals (light gray ribbon). Three vertical dotted lines mark the 4 age groups (before 25, between 25 and 50, between 50 and 75 and above 75 years old). Each point is a protein patient value. The green curve is a Loess regression of the median of ApoA1 values, along with its 95% confidence interval (darker gray). For each age group, the mean difference (%95CI) between patient protein value and the expected normal value (for its age and gender), with its significance p-value is displayed on top of figure. The significance between the non-T2D vs. T2D patients is displayed in green between the 2 panels.

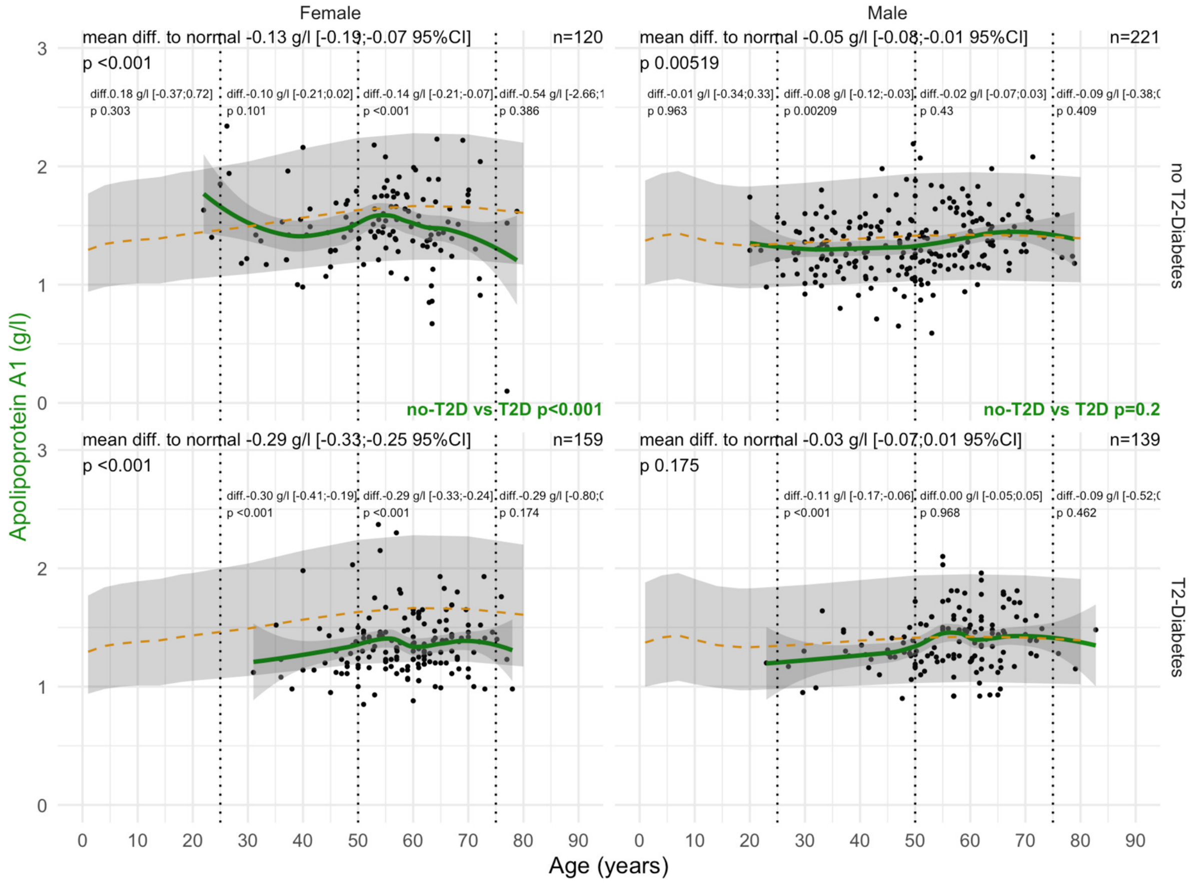

Supplementary Figure S3: ApoA1 NAFLD-Biopsied patients with significant steatosis

Impact of T2D on ApoA1 concentration in NAFD-Biopsied patients with significant steatosis S2S3 (n=693). Normal serum means values by age and gender (dashed orange lines), with 95% confidence intervals (light gray ribbon). Three vertical dotted lines mark the 4 age groups (before 25, between 25 and 50, between 50 and 75 and above 75 years old). Each point is a protein patient value. The green curve is a Loess regression of the median of ApoA1 values, along with its 95% confidence interval (darker gray). For each age group, the mean difference (%95CI) between patient protein value and the expected normal value (for its age and gender), with its significance p-value is displayed on top of figure. The significance between the non-T2D vs. T2D patients is displayed in green between the 2 panels.

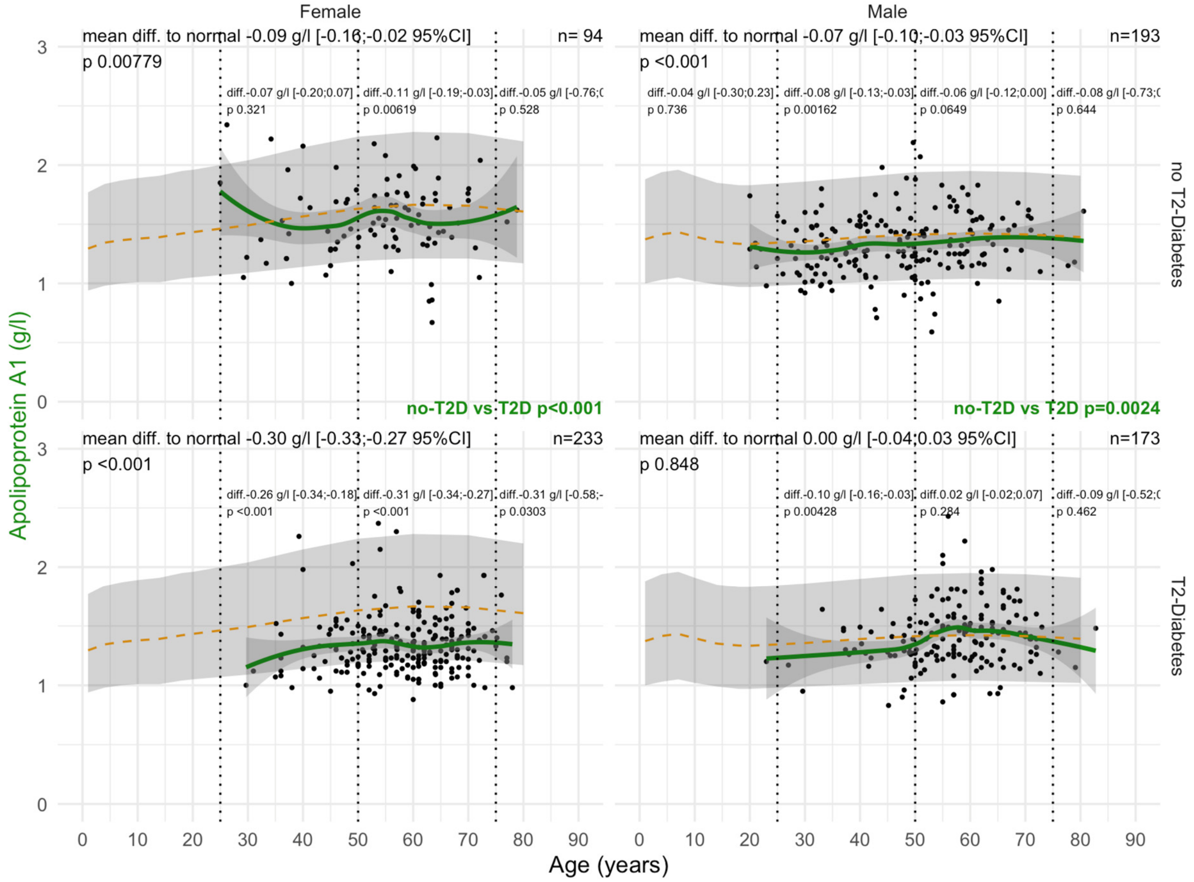

Supplementary Figure S4: ApoA1 in NAFLD-Biopsied patients with obesity

Impact of T2D on ApoA1 concentration in NAFD-Biopsied patients according to obesity (n=495). Normal serum means values by age and gender (dashed orange lines), with 95% confidence intervals (light gray ribbon). Three vertical dotted lines mark the 4 age groups (before 25, between 25 and 50, between 50 and 75 and above 75 years old). Each point is a protein patient value. The green curve is a Loess regression of the median of ApoA1 values, along with its 95% confidence interval (darker gray). For each age group, the mean difference (%95CI) between patient protein value and the expected normal value (for its age and gender), with its significance p-value is displayed on top of figure. The significance between the non-T2D vs. T2D patients is displayed in green between the 2 panels.

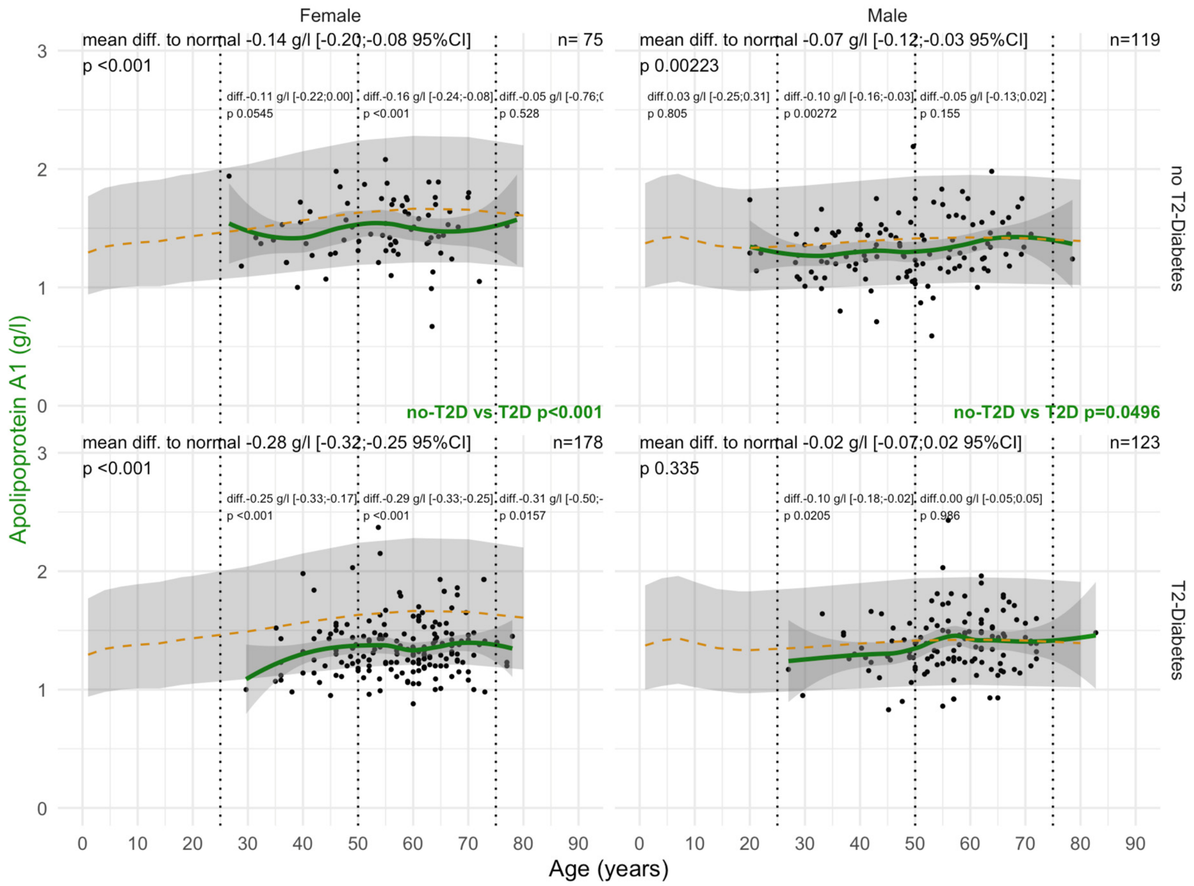

Supplementary Figure S5: A2M in NAFLD-Biopsied patients without obesity

Impact of T2D on A2M concentration in NAFD-Biopsied patients without obesity (n=431). Normal serum means values by age and gender (dashed orange lines), with 95% confidence intervals (light gray ribbon). Three vertical dotted lines mark the 4 age groups (before 25, between 25 and 50, between 50 and 75 and above 75 years old). Each point is a protein patient value. The red curve is a Loess regression of the median of A2M values, along with its 95% confidence interval (darker gray). For each age group, the mean difference (%95CI) between patient protein value and the expected normal value (for its age and gender), with its significance p-value is displayed on top of figure. The significance between the non-T2D vs. T2D patients is displayed in red between the 2 panels.

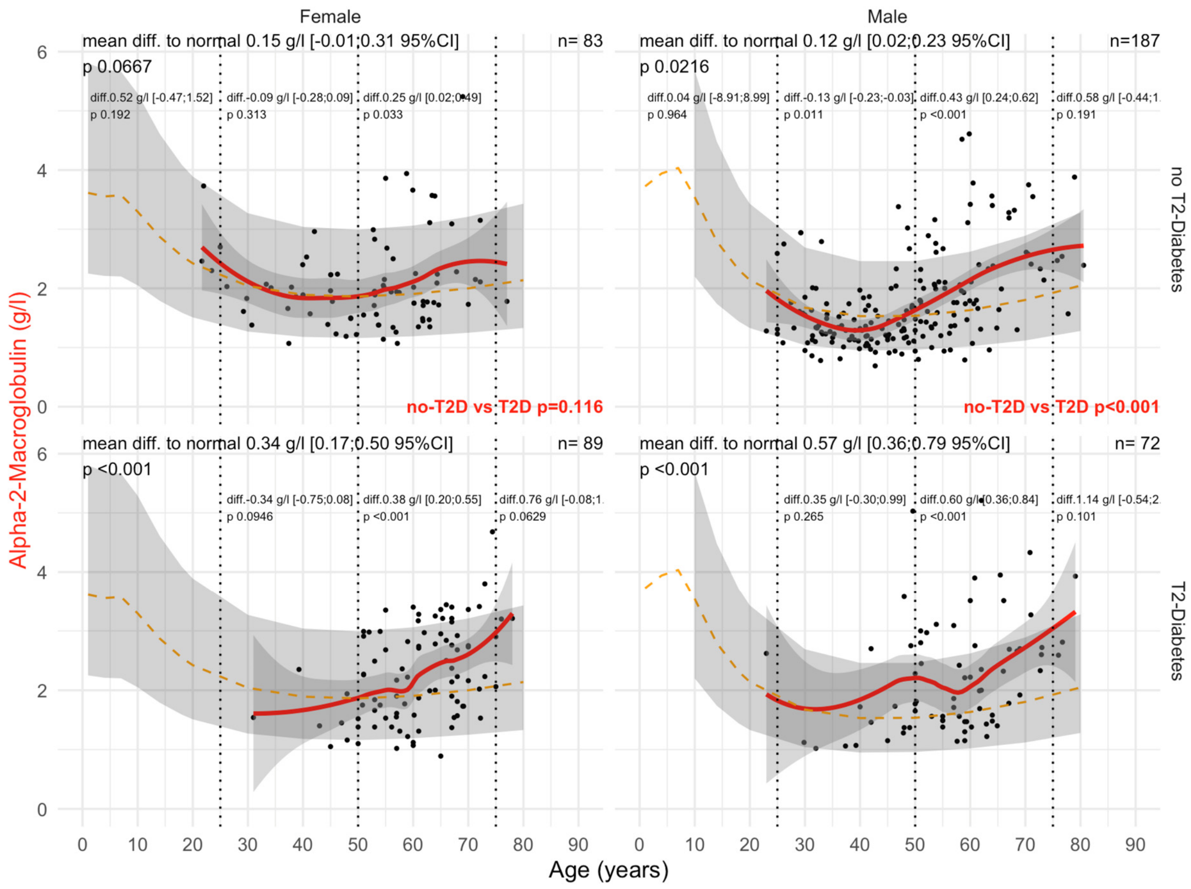

Supplementary Figure S6: A2M in NAFLD-Biopsied patients with obesity

Impact of T2D on A2M concentration in NAFD-Biopsied patients with obesity (n=495). Normal serum means values by age and gender (dashed orange lines), with 95% confidence intervals (light gray ribbon). Three vertical dotted lines mark the 4 age groups (before 25, between 25 and 50, between 50 and 75 and above 75 years old). Each point is a protein patient value. The red curve is a Loess regression of the median of A2M values, along with its 95% confidence interval (darker gray). For each age group, the mean difference (%95CI) between patient protein value and the expected normal value (for its age and gender), with its significance p-value is displayed on top of figure. The significance between the non-T2D vs. T2D patients is displayed in red between the 2 panels.

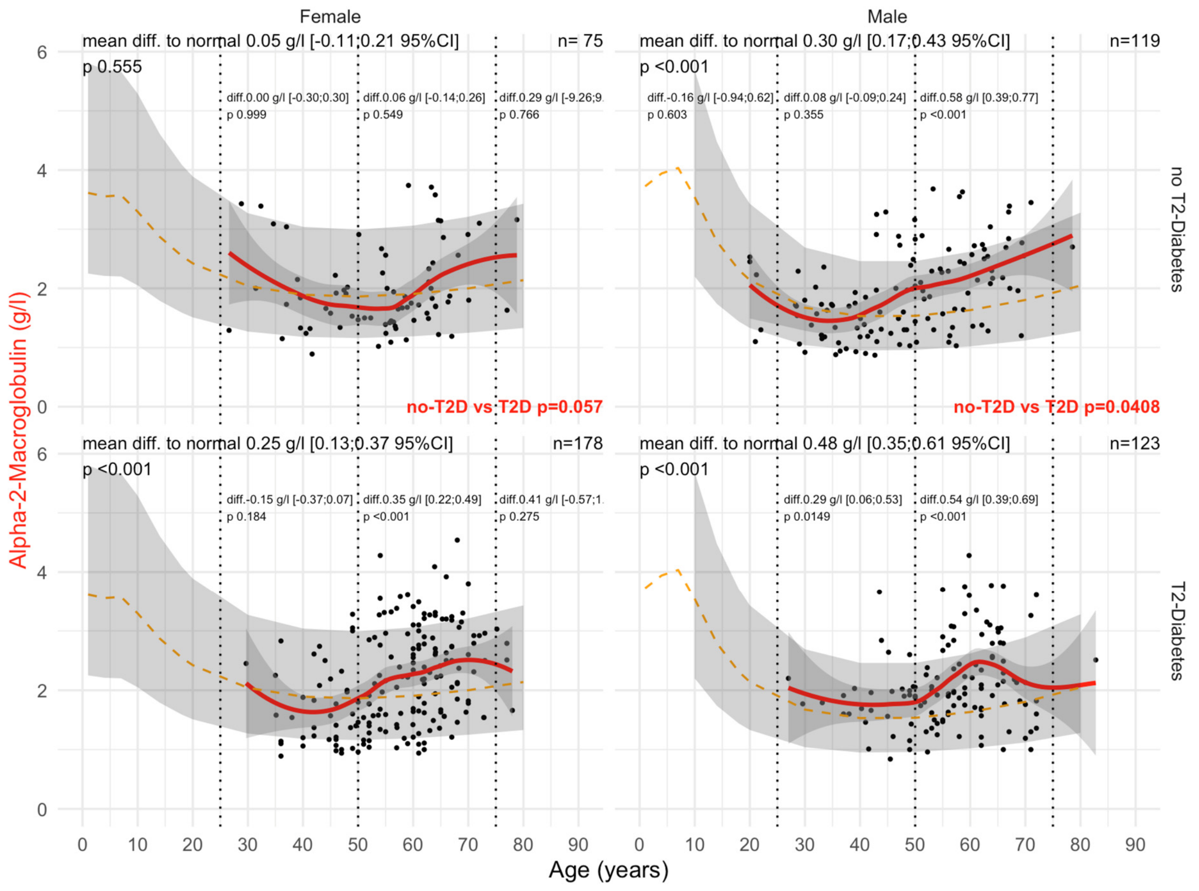

Supplementary Figure S7: ApoA1 in NAFLD-Biopsied patients without obesity

Impact of T2D on ApoA1 concentration in NAFD-Biopsied patients without obesity (n=431). Normal serum means values by age and gender (dashed orange lines), with 95% confidence intervals (light gray ribbon). Three vertical dotted lines mark the 4 age groups (before 25, between 25 and 50, between 50 and 75 and above 75 years old). Each point is a protein patient value. The green curve is a Loess regression of the median of ApoA1 values, along with its 95% confidence interval (darker gray). For each age group, the mean difference (%95CI) between patient protein value and the expected normal value (for its age and gender), with its significance p-value is displayed on top of figure. The significance between the non-T2D vs. T2D patients is displayed in green between the 2 panels.

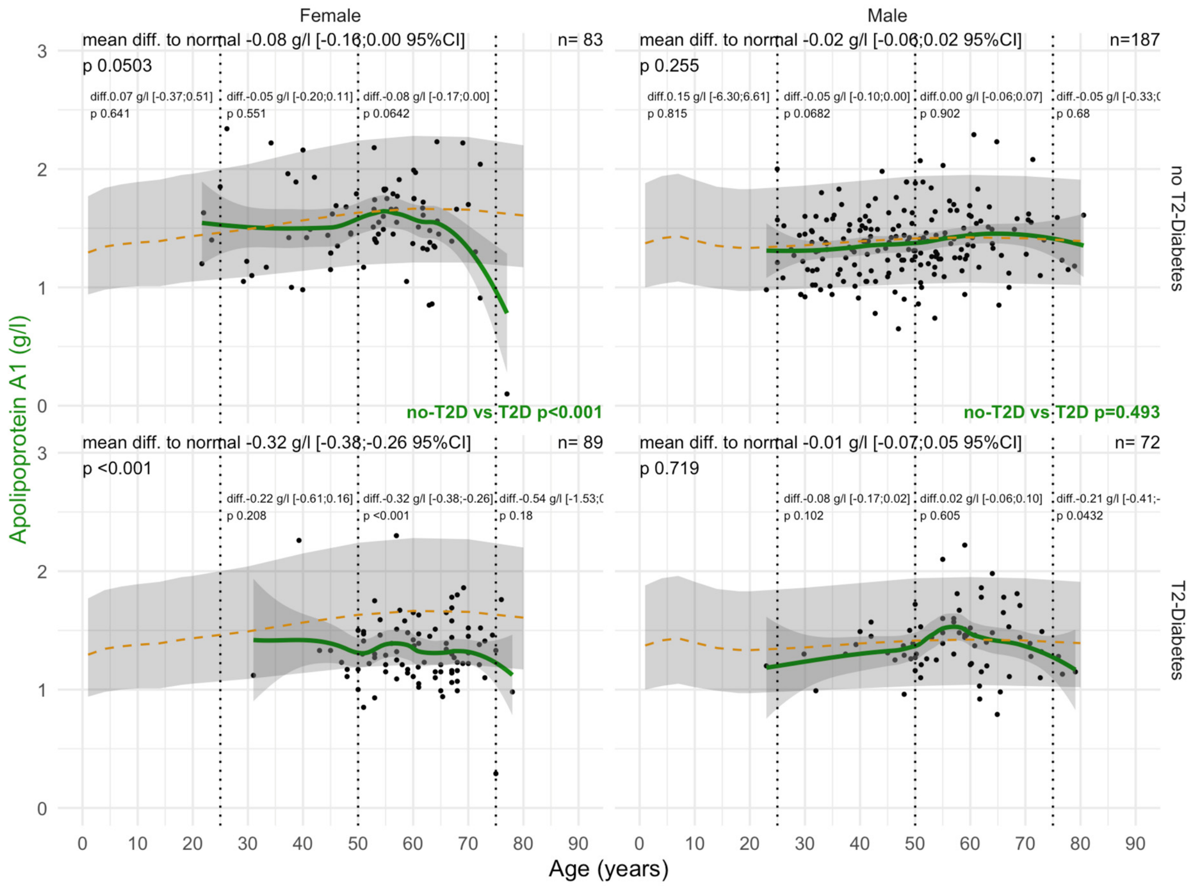

Supplementary Figure S8: Hapto NAFLD-Biopsied patients without obesity

Impact of T2D on Hapto concentration in NAFD-Biopsied patients without obesity (n=431).Normal serum means values by age and gender (dashed orange lines), with 95% confidence intervals (light gray ribbon). Three vertical dotted lines mark the 4 age groups (before 25, between 25 and 50, between 50 and 75 and above 75 years old). Each point is a protein patient value. The blue curve is a Loess regression of the median of Hapto values, along with its 95% confidence interval (darker gray). For each age group, the mean difference (%95CI) between patient protein value and the expected normal value (for its age and gender), with its significance p-value is displayed on top of figure. The significance between the non-T2D vs. T2D patients is displayed in blue between the 2 panels.

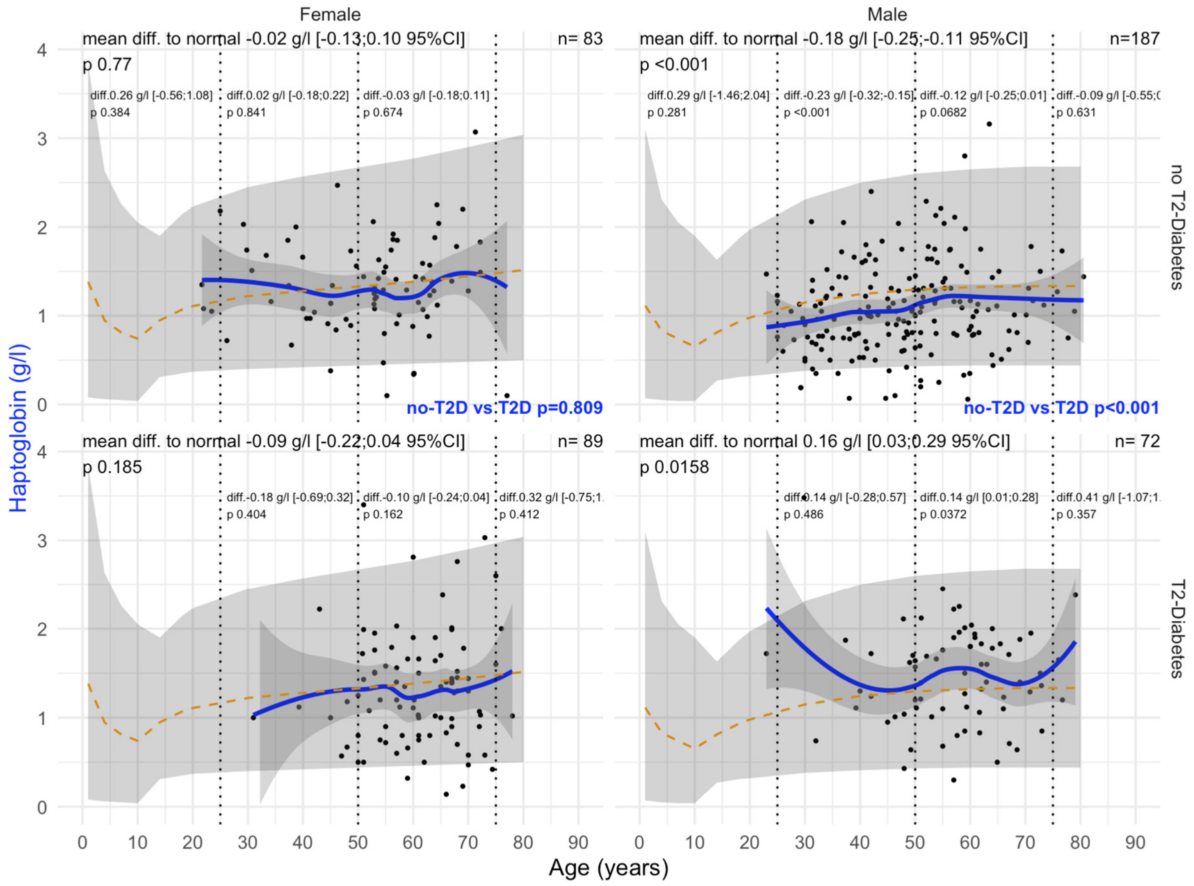

Supplementary Figure S9: Hapto in NAFLD-Biopsied patients with obesity

Impact of T2D on Hapto concentration in NAFD-Biopsied patients with obesity (n=495).Normal serum means values by age and gender (dashed orange lines), with 95% confidence intervals (light gray ribbon). Three vertical dotted lines mark the 4 age groups (before 25, between 25 and 50, between 50 and 75 and above 75 years old). Each point is a protein patient value. The blue curve is a Loess regression of the median of Hapto values, along with its 95% confidence interval (darker gray). For each age group, the mean difference (%95CI) between patient protein value and the expected normal value (for its age and gender), with its significance p-value is displayed on top of figure. The significance between the non-T2D vs. T2D patients is displayed in blue between the 2 panels.

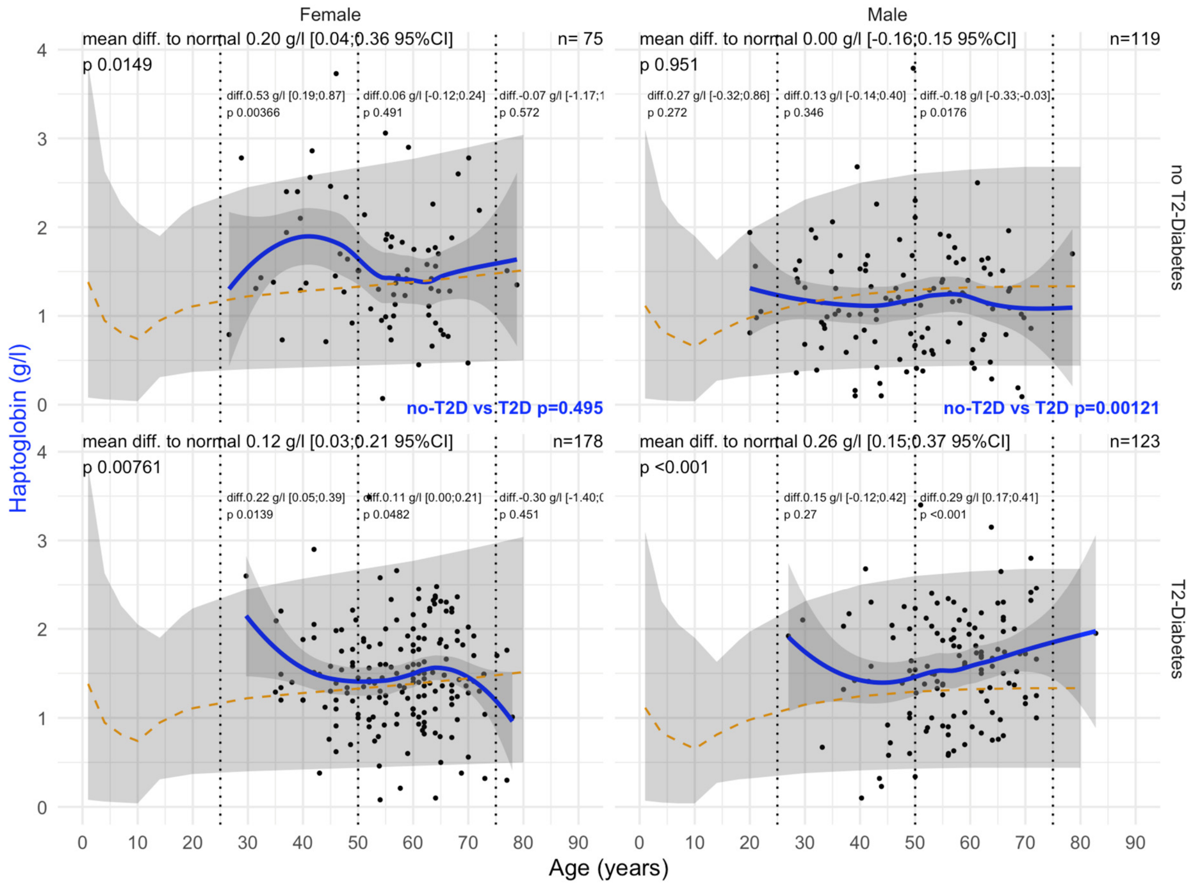

Supplementary Figure S10: Univariate correlations between three proteins and histological features in not-obese et obese male.

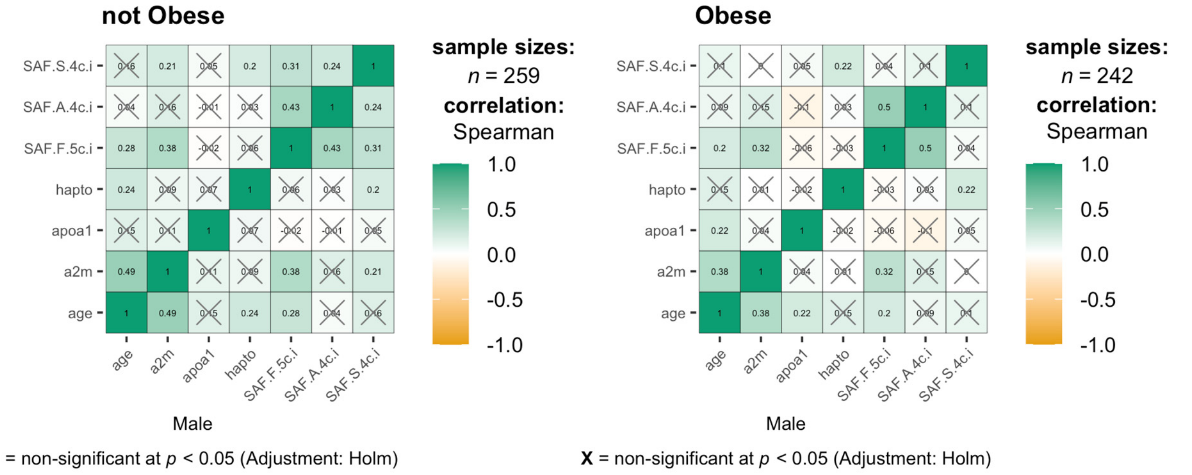

Supplementary Figure S11: Univariate correlations between three proteins and histological features in not-obese et obese female.

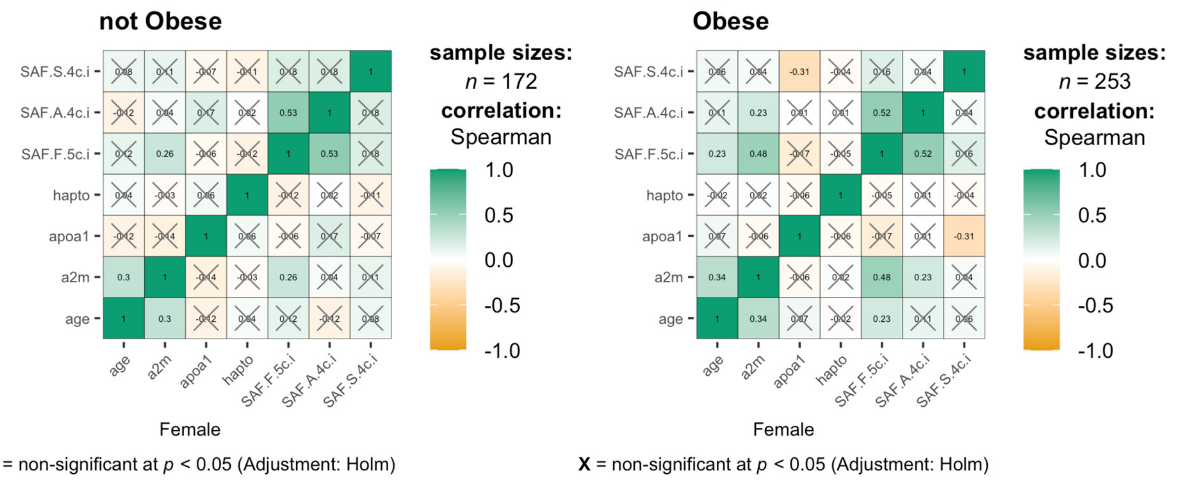

Supplementary Figure S12: Variation of the serum protein levels according to BMI for patients <50 years old

Impact of BMI on the protein concentrations in NAFD-Serum cohort below 50 years old. Median value of the studied group is the horizontal orange dashed line. Five vertical zones mark the 5 BMI groups (WHO definition cut-offs: 18.5, 25, 30, 35 and above 40 kg/m2). Each blue point is a protein patient value with its BMI. The colored curve is a Loess regression of the median of protein values.

Panel A. Alpha-2-Macroglobulin

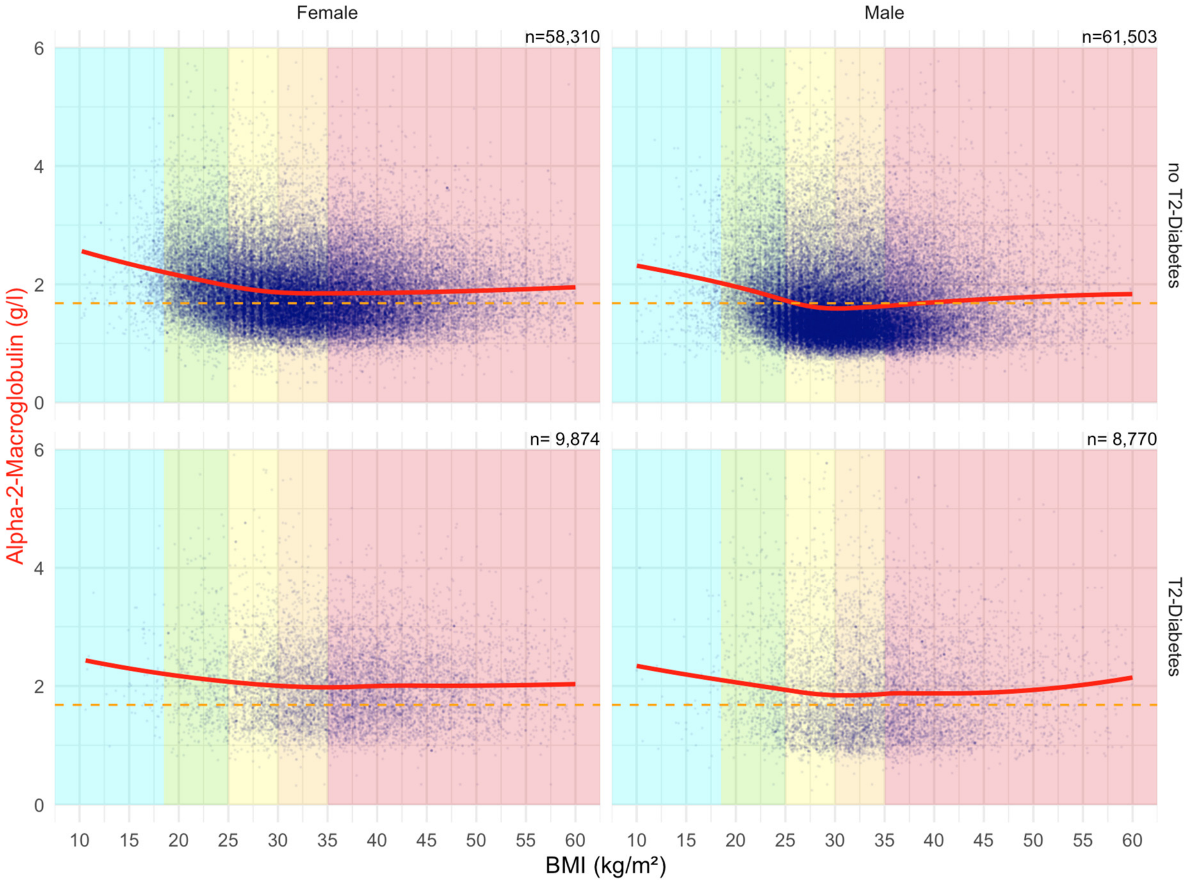

Panel B. Apolipoprotein-A1

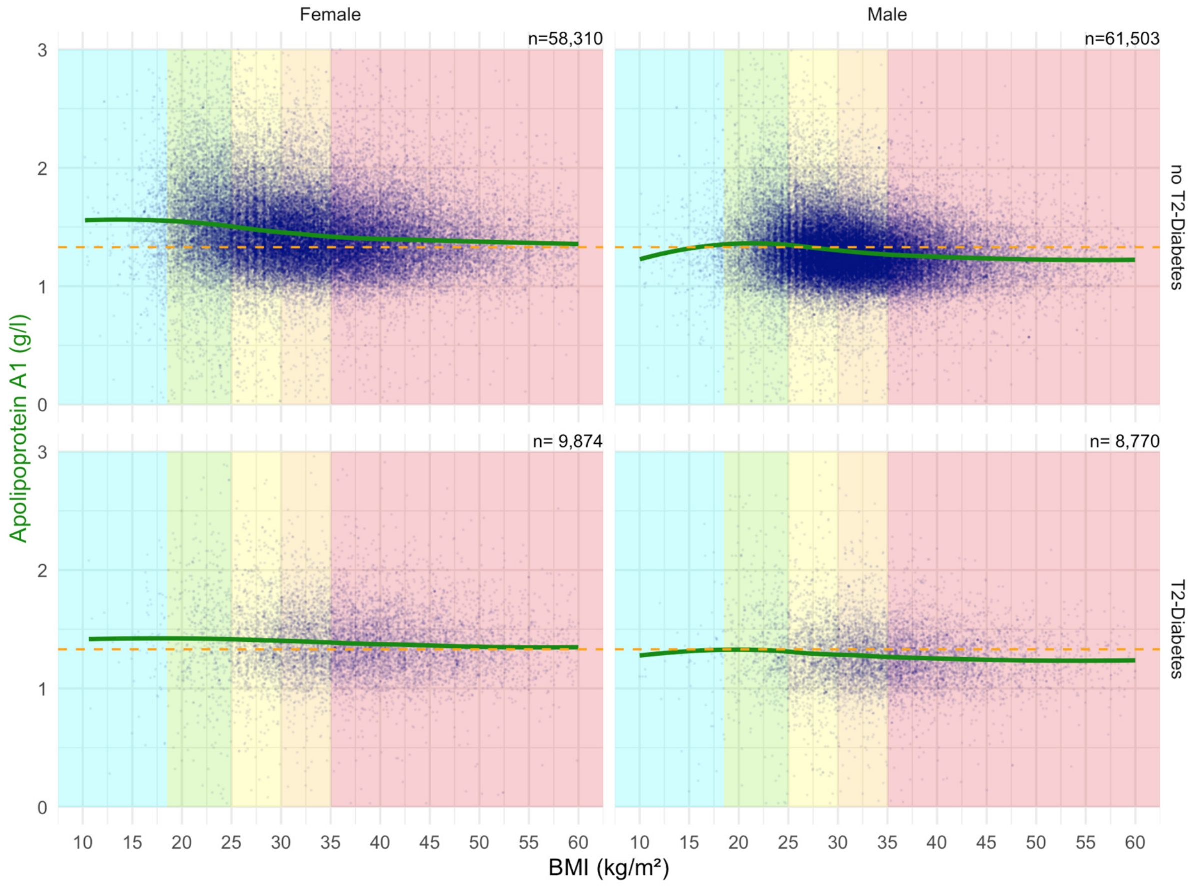

Panel C. Haptoglobin

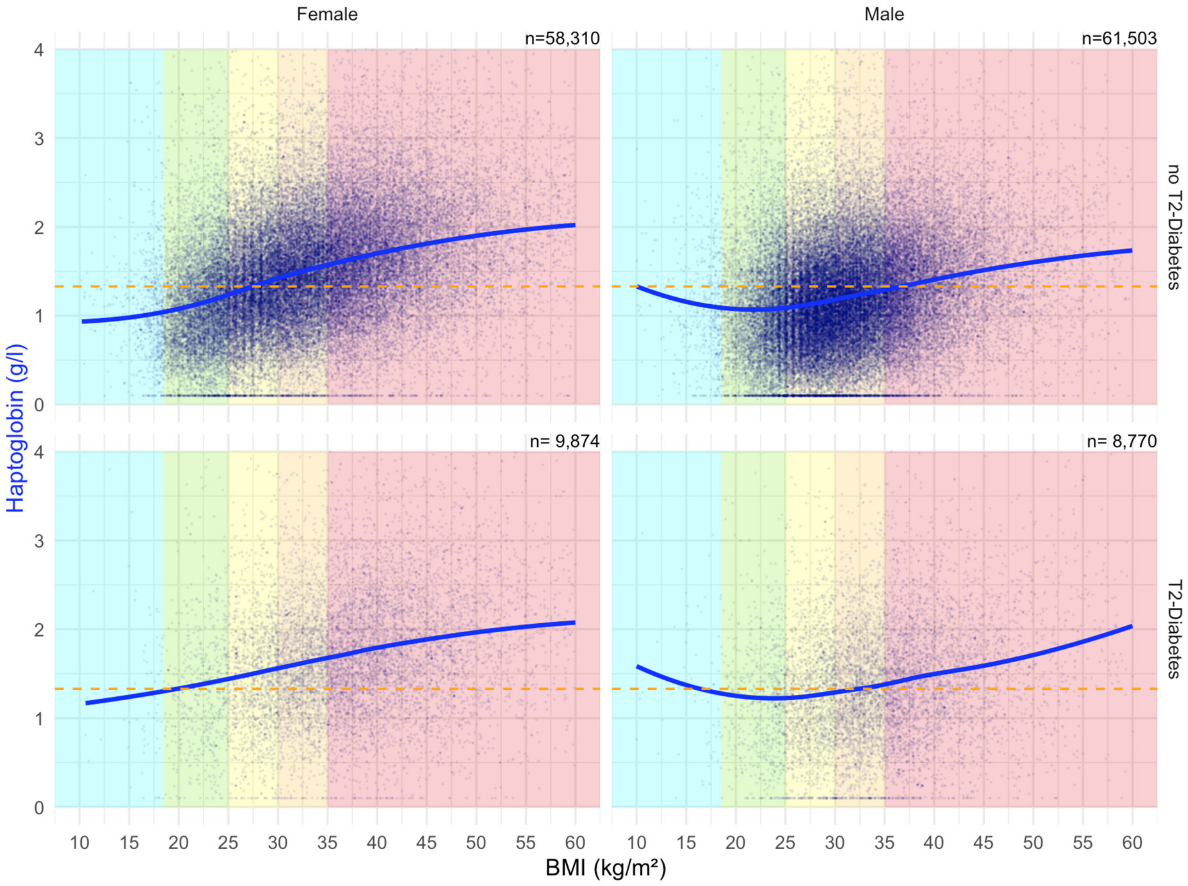

Supplementary Figure S13: Variation of the serum protein levels according to BMI for patients >=50 years old

Impact of BMI on the protein concentrations in NAFD-Serum cohort equal or above 50 years old. Median value of the studied group is the horizontal orange dashed line. Five vertical zones mark the 5 BMI groups (WHO definition cut-offs: 18.5, 25, 30, 35 and above 40 kg/m2). Each blue point is a protein patient value with its BMI. The colored curve is a Loess regression of the median of protein values.

Panel A. Alpha-2-Macroglobulin

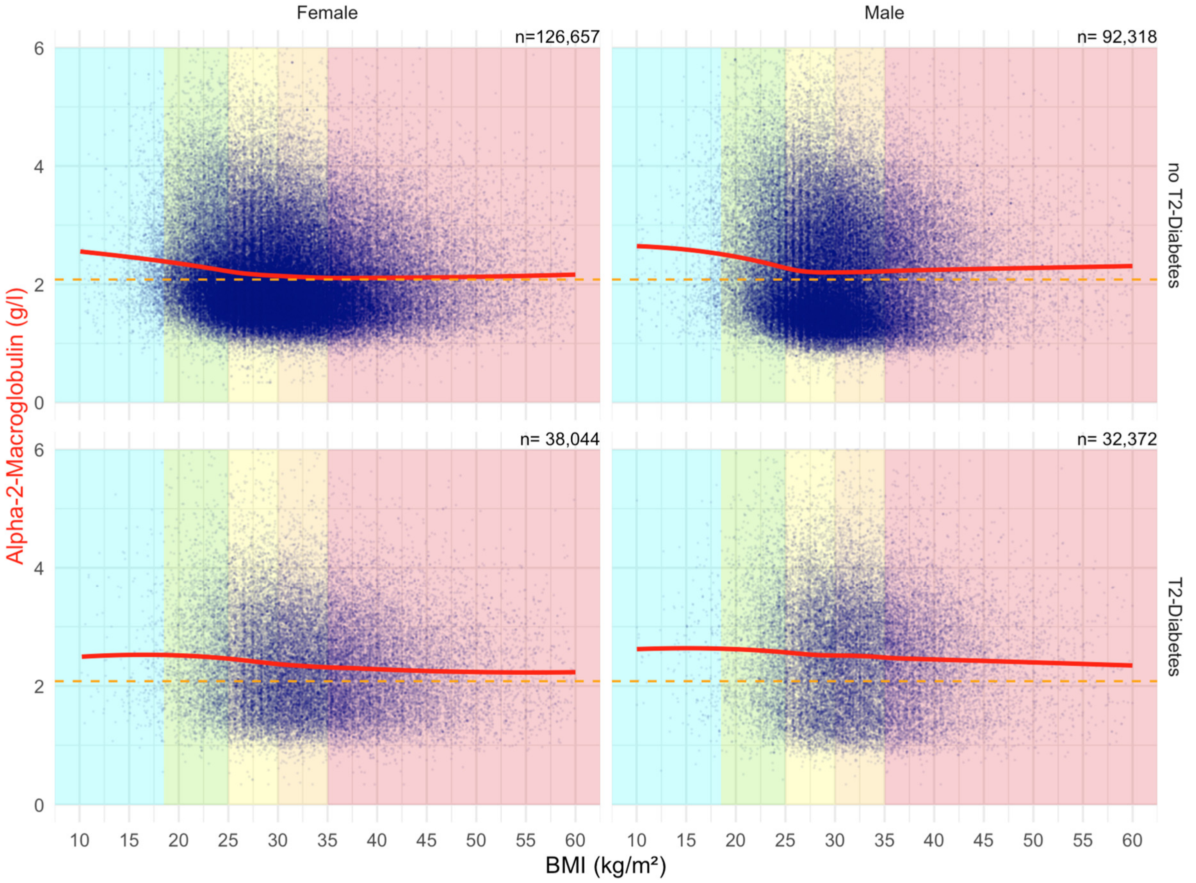

Panel B. Apolipoprotein-A1

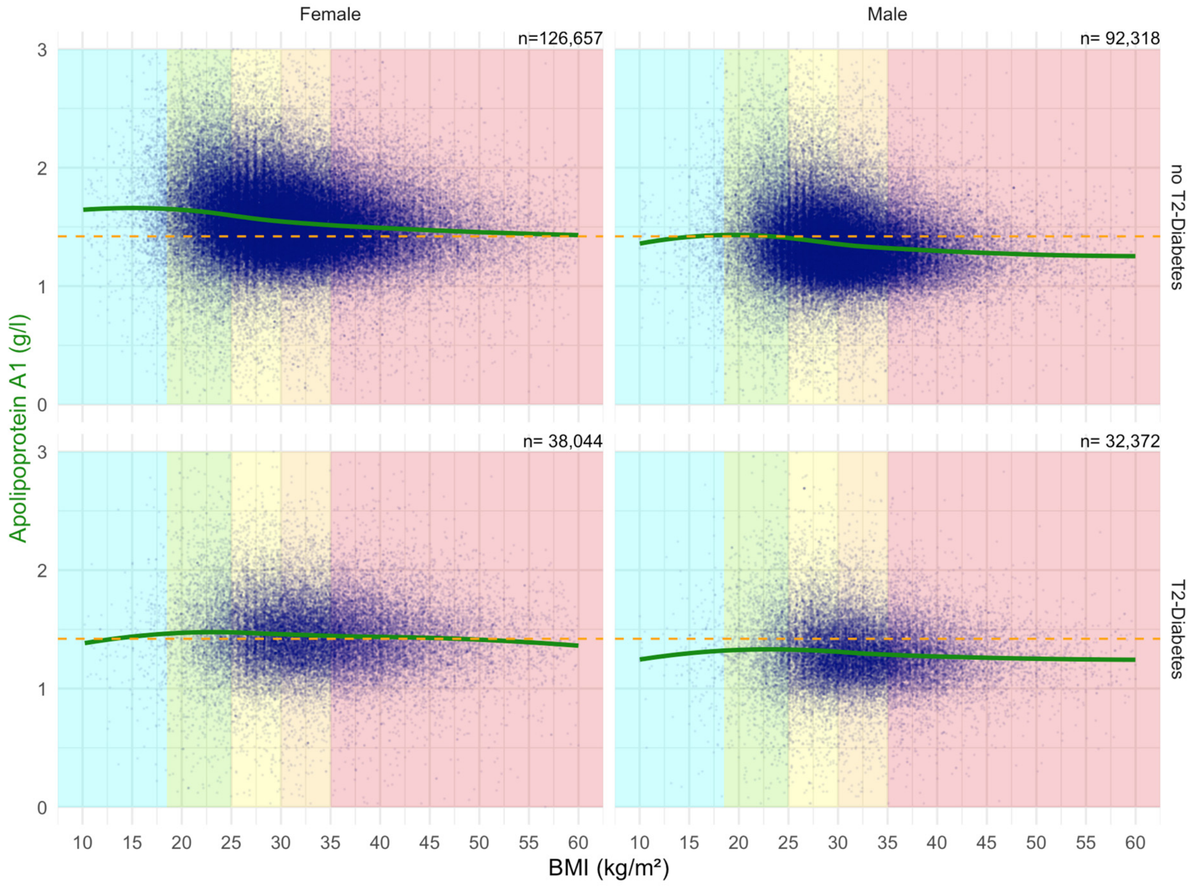

Panel C. Haptoglobin

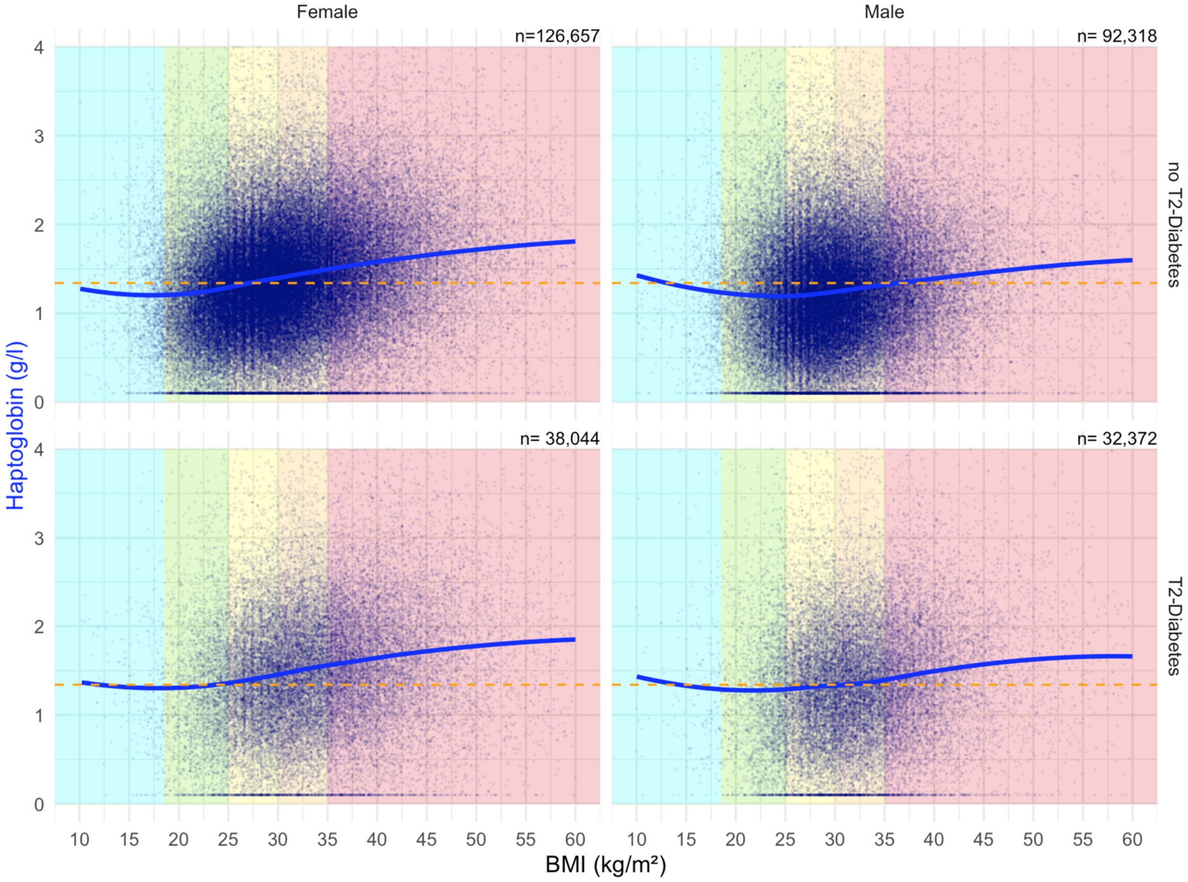

Supplementary Table S1: T2D multivariate association with proteins and fibrosis.

Panel A. Logistic regression in 172 non-obese females, proteins and fibrosis.

Independent variable	Regression coefficient	Standard Error	Wald Z value	P-value	Odds Ratio
Intercept	-1.13620	1.32155	-0.860	0.38993	0.32104
AGE	0.07764	0.01882	4.126	0.00004	1.08073
A2M	-0.01566	0.24707	-0.063	0.94945	0.98446
ApoA1	-2.18938	0.62484	-3.504	**0.00046**	0.11199
HAPTO	-0.05408	0.30561	-0.177	0.85955	0.94736
SAFf5C	-0.02282	0.14643	-0.156	0.87616	0.97744

Panel B. Logistic regression in 253 obese females, proteins and fibrosis.

Independent variable	Regression coefficient	Standard Error	Wald Z value	P-value	Odds Ratio
Intercept	1.07890	1.15212	0.936	0.34904	2.94143
AGE	0.02881	0.01460	1.973	0.04851	1.02923
A2M	0.16856	0.21191	0.795	0.42635	1.18360
ApoA1	-2.06133	0.58713	-3.511	**0.00045**	0.12728
HAPTO	-0.11720	0.23407	-0.501	0.61657	0.88941
SAFf5C	0.03503	0.12863	0.272	0.78538	1.03565

Panel C. Logistic regression in 259 non-obese males, proteins and fibrosis.

**Independent Variable**	**Regression Coefficient**	**Standard Error**	**Wald Z-Value**	**P-value**	**Odds Ratio**
Intercept	-4.94771	1.11813	-4.425	0.00001	0.00710
AGE	0.03733	0.01478	2.526	0.01155	1.03804
A2M	0.24153	0.20476	1.180	0.23816	1.27320
ApoA1	0.12632	0.56403	0.224	0.82279	1.13464
HAPTO	1.27345	0.29571	4.306	**0.00002**	3.57317
SAFf5C	0.45971	0.15081	3.048	0.00230	1.58361

Panel D. Logistic regression in 242 obese males, proteins and fibrosis.

Independent Variable	Regression Coefficient	Standard Error	Wald Z-Value	P-value	Odds Ratio
Intercept	-4.41602	0.98833	-4.468	0.00001	0.01208
AGE	0.05999	0.01397	4.295	0.00002	1.06183
A2M	0.10995	0.21928	0.501	0.61607	1.11622
ApoA1	0.44088	0.56351	0.782	0.43399	1.55408
HAPTO	0.52823	0.20628	2.561	**0.01044**	1.69592
SAFf5C	-0.15541	0.12809	-1.213	0.22500	0.85606

Supplementary Table S2. T2D multivariate association with proteins and NASH.

Panel A. Logistic regression in 172 non-obese females, proteins and NASH.

Independent variable	Regression coefficient	Standard Error	Wald Z value	P-value	Odds Ratio
Intercept	-0.62552	1.34869	-0.464	0.64279	0.53498
AGE	0.07353	0.01873	3.926	0.00009	1.07630
A2M	0.06665	0.23916	0.279	0.78050	1.06892
ApoA1	-2.01859	0.63580	-3.175	**0.00150**	0.13284
HAPTO	-0.06572	0.31147	-0.211	0.83288	0.93639
SAFa4C	-0.40117	0.16177	-2.480	0.01314	0.66954

Panel B. Logistic regression in 253 obese females, proteins and NASH.

Independent variable	Regression coefficient	Standard Error	Wald Z value	P-value	Odds Ratio
Intercept	1.57295	1.18511	1.327	0.18442	4.82085
AGE	0.03261	0.01489	2.190	0.02854	1.03315
A2M	0.32291	0.20227	1.596	0.11040	1.38114
ApoA1	-2.12433	0.59745	-3.556	**0.00038**	0.11951
HAPTO	-0.12828	0.23334	-0.550	0.58249	0.87961
SAFa4C	-0.41350	0.14688	-2.815	0.00488	0.66133

Panel C. Logistic regression in 259 non-obese males, proteins and NASH.

**Independent Variable**	**Regression Coefficient**	**Standard Error**	**Wald Z-Value**	**P-value**	**Odds Ratio**
Intercept	-4.66734	1.11357	-4.191	0.00003	0.00940
AGE	0.04033	0.01426	2.828	0.00469	1.04116
A2M	0.43500	0.19868	2.189	**0.02856**	1.54496
ApoA1	0.06746	0.55234	0.122	0.90280	1.06978
HAPTO	1.23179	0.29054	4.240	**0.00002**	3.42736
SAFa4C	0.01719	0.14666	0.117	0.90672	1.01733

Panel D. Logistic regression in 242 obese males, proteins and NASH.

Independent Variable	Regression Coefficient	Standard Error	Wald Z-Value	P-value	Odds Ratio
Intercept	-4.07480	1.02270	-3.984	0.00007	0.01700
AGE	0.05916	0.01400	4.225	0.00002	1.06095
A2M	0.07969	0.21217	0.376	0.70721	1.08296
ApoA1	0.38948	0.56545	0.689	0.49095	1.47622
HAPTO	0.54754	0.20654	2.651	**0.00803**	1.72899
SAFa4C	-0.23614	0.14663	-1.610	0.10729	0.78967

Supplementary Table S3. T2D multivariate association with proteins and steatosis.

Panel A. T2D association in 172 non-obese females, with proteins and steatosis.

Independent variable	Regression coefficient	Standard Error	Wald Z value	P-value	Odds Ratio
Intercept	-4.17680	1.62374	-2.572	0.01010	0.01535
AGE	0.08766	0.02187	4.008	0.00006	1.09161
A2M	-0.10632	0.27488	-0.387	0.69891	0.89914
ApoA1	-2.36502	0.68935	-3.431	**0.00060**	0.09395
HAPTO	0.26817	0.34425	0.779	0.43597	1.30758
SAFsC	1.29956	0.26246	4.952	0.00000	3.66768

Panel B. T2D association in 253 obese females, with proteins and steatosis.

Independent variable	Regression coefficient	Standard Error	Wald Z value	P-value	Odds Ratio
Intercept	-3.48254	1.44147	-2.416	0.01569	0.03073
AGE	0.02295	0.01623	1.414	0.15750	1.02321
A2M	0.21597	0.21446	1.007	0.31392	1.24106
ApoA1	-1.01572	0.63999	-1.587	0.11250	0.36214
HAPTO	-0.08845	0.24561	-0.360	0.71875	0.91535
SAFsC	1.66506	0.26615	6.256	0.00000	5.28599

Panel C. T2D association in 259 non-obese males, with proteins and steatosis.

**Independent Variable**	**Regression Coefficient**	**Standard Error**	**Wald Z-Value**	**P-value**	**Odds Ratio**
Intercept	-6.36934	1.23455	-5.159	0.00000	0.00171
AGE	0.04080	0.01529	2.669	0.00760	1.04164
A2M	0.30442	0.20867	1.459	0.14460	1.35584
ApoA1	-0.36782	0.63161	-0.582	0.56034	0.69224
HAPTO	1.07896	0.31214	3.457	**0.00055**	2.94162
SAFsC	1.48949	0.28673	5.195	0.00000	4.43484

Panel D. T2D association in 242 obese males, with proteins and steatosis.

Independent Variable	Regression Coefficient	Standard Error	Wald Z-Value	P-value	Odds Ratio
Intercept	-9.72924	1.42535	-6.826	0.00000	0.00006
AGE	0.06457	0.01573	4.105	0.00004	1.06670
A2M	0.13640	0.24390	0.559	0.57599	1.14614
ApoA1	0.54740	0.63772	0.858	0.39069	1.72875
HAPTO	0.32084	0.20883	1.536	0.12446	1.37828
SAFsC	2.39136	0.38616	6.193	0.00000	10.92830
